# Individual and Combined Application of Nematophagous Fungi as Biological Control Agents against Gastrointestinal Nematodes in Domestic Animals

**DOI:** 10.3390/pathogens11020172

**Published:** 2022-01-27

**Authors:** Shuoshuo Li, Da Wang, Jianchuan Gong, Ying Zhang

**Affiliations:** 1 State Key Laboratory for Conservation and Utilization of Bio-Resources in Yunnan, Key Laboratory for Southwest Microbial Diversity of the Ministry of Education, Yunnan University, Kunming 650032, China; lishuoshuo@mail.ynu.edu.cn (S.L.); wangdawangda@mail.ynu.edu.cn (D.W.); gongyusheng@mail.ynu.edu.cn (J.G.); 2 School of Life Science, Yunnan University, Kunming 650032, China

**Keywords:** predatory fungi, ovicidal fungi, gastrointestinal nematodes (GINs), chemical anthelmintic drugs, nematicidal activity

## Abstract

Gastrointestinal nematodes (GINs) are a group of parasites that threaten livestock yields, and the consequent economic losses have led to major concern in the agricultural industry worldwide. The high frequency of anthelmintic resistance amongst GINs has prompted the search for sustainable alternatives. Recently, a substantial number of both in vitro and in vivo experiments have shown that biological controls based on predatory fungi and ovicidal fungi are the most promising alternatives to chemical controls. In this respect, the morphological characteristics of the most representative species of these two large groups of fungi, their nematicidal activity and mechanisms of action against GINs, have been increasingly studied. Given the limitation of the independent use of a single nematophagous fungus (NF), combined applications which combine multiple fungi, or fungi and chemical controls, have become increasingly popular, although these new strategies still have antagonistic effects on the candidates. In this review, we summarize both the advantages and disadvantages of the individual fungi and the combined applications identified to date to minimize recurring infections or to disrupt the life cycle of GINs. The need to discover novel and high-efficiency nematicidal isolates and the application of our understanding to the appropriate selection of associated applications are discussed.

## 1. Introduction

Nematodes are the most abundant animals on Earth, with approximately 4.4 ± 0.64 × 10^20^ individuals inhabiting the upper layer of soils across the globe [1,2]. The species diversity of the terrestrial nematode community is high, but most members lack the ability to decompose organic matter [3]. Thus, feeding habits are fundamental in their nutrient cycles and provide the basis for definitions of the essential feeding types of terrestrial nematodes. The following groups of hosts are recognized: animals, plants, fungi, bacteria, and unicellular eukaryotes [4]. With professional helminths being among them, animal- and plant-parasitic nematodes contribute to great economic losses to agriculture worldwide. Gastrointestinal helminth parasites, which are harmful to animal health, have a significant impact on the productivity and quality of livestock [5,6,7]. The successful control of helminths relies heavily on their disruption across the entire life cycle.

**Figure 1:**
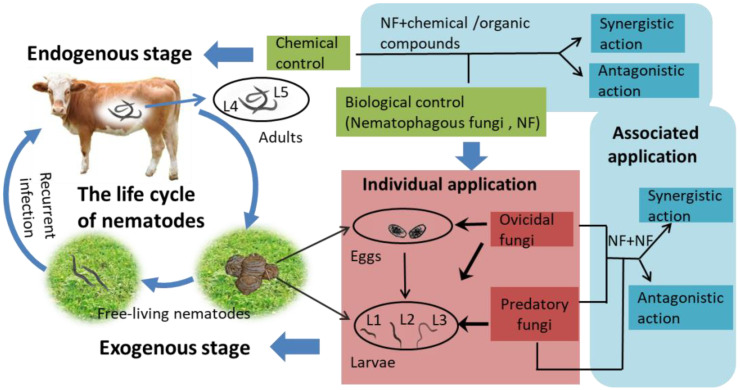
The life cycle of gastrointestinal nematodes (GINs) includes exogenous and endogenous stages. The application of individual fungi is used to eliminate exogenous nematodes, and the combined application of multiple fungi or fungi and chemical controls is used to eliminate endogenous nematodes.

The life cycle of gastrointestinal parasitic nematodes in animals consists of two stages: (1) in the endogenous stage, the adults (the fourth or fifth larval stage; L4/L5) are parasitic in the gastrointestinal tracts (GT) of animals, and (2) in the exogenous stage, the infected adults are excreted from the animal and develop into eggs and larvae in the feces, where the larvae go through their first and second larval stages and enter the infective third larval stage (L3); subsequently, free-living nematodes (eggs, larvae, cysts) integrate into pasture and are ingested by livestock during grazing, which leads to recurrent infection by nematodes [8,9,10,11] (Figure 1). As a matter of fact, epidemiological studies have indicated that only 5% of gastrointestinal nematodes (GINs) are located within animals, while 95% live on pasture in the form of eggs and larvae [12,13,14]. Therefore, it is necessary to focus on the control of nematodes at the exogenous stage so as to minimize recurrent infections [15,16,17]. In order to control nematodes more efficiently, it is important to interfere with their entire life cycle—that is, to reduce the numbers of nematodes in animals and on the pasture at the same time.

Chemical controls are the traditional method used for reducing GINs, but the frequently reported disadvantages of this method include the development of resistance in the nematodes and the potential risk to human health from nematode contamination of the environment via the residue in animal products and feces [18,19,20]. However, nematophagous fungi (NF), as natural enemies of gastrointestinal helminth parasites, shape as the alternative to chemical controls with the most potential; they can be used to control the immature nematodes present in animal feces [21,22]. In recent years, biological controls have become an important research area in the control of helminths due to the absence of the disadvantages of chemical controls and conformity to the goals of ecological sustainability [23,24,25]. Predatory and ovicidal NF fungi have been shown in extensive in vitro and in vivo tests to effectively reduce recurrent infections by GINs in domestic animals [26]. However, in order to further enhance nematicidal activity, current studies now employ new strategies with combined applications of multiple fungi or fungi and chemical controls to minimize recurring infections or to disrupt the whole life cycle of nematodes [27] (Figure 1). Until recently, individual agent application was the mainstream solution to controlling nematode populations, while combined applications were an auxiliary method, because the incompatibility and inefficiency of the latter could not be ignored.

This review concentrates on the latest research in which NF are viewed as the most promising biocontrol agents of GINs in domestic animals. We have collected and commented on the literature related to combined applications and explained that such a strategy can have an additive effect under some circumstances. Aiming at the application of NF for domestic animal GIN infection, this review outlines the need to discover novel and high-efficiency nematicidal isolates and the need to choose a combined application when application of a single solution is ineffective. This new strategy may serve as a complementary method for biological control and provide a new research direction for future GIN management.

## 2. The Most Representative Biocontrol Candidates—Individual Application

NF are divided into five groups: predatory, opportunistic or ovicidal, endoparasitic, toxin-producing, and producers of special attack devices [28,29], but most studies on the biological control of GINs have been restricted to the two traditional groups of NF: predatory fungi and ovicidal fungi. To date, numerous experiments, both in vivo and in vitro, have verified that the representative species of the two NF have common advantages in individual application: (1) no loss of predatory viability as they pass through the GTs of animals; (2) the effective reduction of immature-stage nematodes, including eggs and larvae; (3) a strong ability to germinate spores in feces. 

### 2.1. Predatory Fungi

Predatory fungi are a key tool in killing nematodes. They produce modified hyphae called traps, which can be either adhesive trapping devices (network, hyphae, branches, knobs) or non-adhesive trapping devices (non-constricting or constricting rings) [30,31]. These traps bind and digest both adult and larval nematodes via mechanical or enzymatic processes [32]. Lysek and Araújo et al. found that this group of fungi only showed physiological effects; hyphae adhered to the eggshells of helminths without egg destruction [33,34]. Therefore, the use of these fungi may have low efficiency, because undamaged eggs can cause recurrent infections [24]. There is, however, no doubt that the effect of predatory fungi on the larvae of GINs is satisfactory. According to the most recent taxonomic classification, all predatory orbiliaceous fungi are assigned into three genera—*Arthrobotrys*, *Drechsleralla*, and *Dactylellina*—based on the use of trapping devices as the primary criterion for generic delimitation [35,36]. However, the old species names also appeared in the literature studied in this review.

#### 2.1.1. Duddingtonia

The genus *Duddingtonia* possesses an outstanding characteristic in that it produces numerous chlamydospores, a type of thick-walled spore [37]. These spores have a shape that can range from elliptical to ovoid with a median septum [38]. The production of abundant chlamydospores is an advantage, because abundant fungal spores are an important strategy for survival and spread. Consequently, this group of fungi is more successful than other genera in the control of nematodes [39].

*Duddingtonia flagrans* is one of the most widely studied and most promising species. Its chlamydospores can withstand gastrointestinal transportation and other undesirable environments to germinate, forming as a predator device a three-dimensional network structure to capture living larvae in animal feces [37,40]. Experimental studies in vitro have shown that *D. flagrans* could reduce up to 96.4% of GINs, better than *Monacrosporium thaumasium* and *Arthrobotrys robusta* [41,42], and in in vivo tests a reduction of 55.15%–98.82% has been reported [43]. With the development of next-generation sequencing, genomic analysis of *D. flagrans* has shown that the species contains more abundant genes relating to the pathogenicity of nematodes than other fungi, such as cytochrome P450 genes and protease-coding genes, which provide *D. flagrans* with stronger nematicidal activity and keep other enemies (such as fungal-feeding nematodes) from feeding [44,45]. Moreover, *D. flagrans* has fewer carbohydrate-degradation-related genes and a weaker saprophytic capability than other fungi, which makes *D. flagrans* rely on nematodes as its sole nutrition source, which is associated with its excellent ability to form traps [44,46]. To date, *D. flagrans* has been viewed as a good controller of trichostrongylides and cyathostome (the most prevalent GINs in livestock) in various domestic animals (Table 1). Bioverm®, a fungal formulation that contains chlamydospores of *D. flagrans*, has been licensed for commercialization in Brazil [17,47].

#### 2.1.2. Arthrobotrys

*Arthrobotrys* is a typical genus of NF and was the first discovered in the 19th century [56]. This genus is characterized by a high ability to produce conidia and chlamydospores, an innate advantage that is not present in all NF [48]. *Arthrobotrys* has been deemed one of the most important genera to be used as a potential biocontrol agent among the predatory fungi to date. Conidiophores of species in this genus are typically simple or sparingly branched, bearing apical clusters of conidia [57,58].

Table 2 shows that some species of the genus, including *A. conoides*, *A. sinense*, *A. musiformis*, and *A. robusta*, reduced 80%–99% of GINs in in vitro studies. Although these species of the genus showed high efficiency for trichostrongylides, the percentage reduction of nematodes was different at the same dose. In addition to these species, *A. oligospora*, *A. cladodes*, and *A. superba* reduced the larvae of *Haemonchus* sp. by up to 90%. Importantly, in in vivo studies, all these species were effective in reducing larvae by approximately 53%–97%.

Generally, NF can be made into edible pellets, mixed with grass, and then fed into the GT through animal chewing. During this series of processes, chewing causes mechanical damage to NF, and GT physical and chemical factors also have an impact on them [70]. However, in vivo experiments have proven that several species of *Arthrobotrys* can reach animal feces smoothly through the GT and function successfully.

In contrast to *D. flagrans*, many species of *Arthrobotrys* have shown effectiveness against trichostrongylides and cyathostomes in various livestock. However, *Arthrobotrys* is more saprophytic than *D. flagrans*, so it cannot spontaneously form traps without the induction of nematodes, amino acids, or environmental conditions [71].

In short, multiple species in this genus could serve as potential candidates for GIN control.

#### 2.1.3. Monacrosporium

Fungi of the *Monacrosporium* genus have a well-developed ability to produce chlamydospores and form traps on conidia or germlings [30]. The genus is defined by a single conidium produced on each tip of the conidiophores [72].

Importantly, the genus can still survive and maintain nematicidal ability after passing through the GTs of animals, which is a prerequisite for fungi to act on nematodes in feces [73]. *Mo. thaumasium* has been successfully used in laboratories and under field conditions in the control of GINs in domestic animals (Table 1). Moreover *Mo. thaumasium* acts as a cooperator in combination with other fungi to kill nematodes, and has been shown to improve the efficiency of the predation of nematodes to some extent [74].

As the mycelium of *Mo. Thaumasium* grows slowly, it needs to be processed before application in experiments. For example, *Panagrellus* sp. can stimulate the growth of the mycelium of the genus *Monacrosporium* [53], but this kind of pretreatment is rare. On the other hand, much like *D. flagrans*, *Mo. Thaumasium* cannot destroy the eggs of GINs [19]. It is important to discover other fungal species with a strong ability to kill sedentary nematodes (females and eggs).

### 2.2. Ovicidal Fungi

Ovicidal fungi are common soil saprophytes, and are opportunistic isolates obtained from the sedentary stages (female and egg stages) of sedentary nematodes [26,57]. The majority of isolated ovicidal fungi have been found to belong to *Humicola*, *Pochonia*, *Martiellera*, *Paecilomyces*, and *Fusarium* [58]. Over the years, *Pochonia* has been treated as the most representative genus, with significant ovicidal action reported for GINs [26]. Unlike predatory fungi, this group of fungi cannot form trapping devices [75]. Their hyphal penetration and internal egg colonization operates via a mechanical/enzymatic process, with morphological changes in the eggshell and embryo observed [22]. However, the larvicidal activity of ovicidal fungi is rarely evaluated.

#### 2.2.1. Pochonia

*Pochonia* belongs to the *Hypocreales* order (Ascomycota). In the ovicidal fungal (opportunist) group, *Pochonia chlamydosporia* (previously known as *Verticillium chlamydosporium*) stands out [54]. This species is used to form dictyochlamydospores and has been extensively studied as a biocontrol agent [76].

*Po. chlamydosporia* selectively parasitizes the eggs of gastrointestinal helminths and females. Its appressoria can not only colonize the surfaces of eggs, but also penetrate into the insides of the eggs in the process of fungal action on nematodes [76,77]. To date, *Po. chlamydosporia* has been used as the most common ovicidal fungi to control GINs in various domestic animals, and it has been shown to reduce nematode eggs by 87.4% in in vitro tests (Table 2). It is worth mentioning that *Pochonia* are inoffensive to animals and humans [63].

Although the ovicidal activity of *Po. chlamydosporia* has been frequently evaluated, it is not known whether it has destructive power against larvae. Vieira et al. specifically studied the ability of *Po. chlamydosporia* to capture larvae and found that it reduced 66.8% of L3 GINs in cattle [78], but there are few reports in the literature on the larvicidal activity of this fungus.

#### 2.2.2. Other

It has been reported that other genera of NF, including *Paecilomyces* and *Mucor*, also have ovicidal action.

*Paecilomyces lilacinus* is a common hyphomycete which has proven efficiency on the eggs of gastrointestinal parasite nematodes and tapeworms in ruminants and human beings [79,80]. It has been found to be able to reduce the number of nematode eggs in the feces of dogs and horses (Table 2), but the effect was not as good as that of *Po. chlamydosporia* [60,67]. The fungus presents a safety risk for humans and animals because it can produce neutral straight-chain peptide toxins (paecilotoxins) [81,82]. As a result, *Paecilomyces* should be regarded more as a fungicide than as a researched genus.

*Mucor circinelloides*, a soil filamentous fungus, is able to destroy nematode eggs in the feces of infected animals [83]. In the presence of the eggs of ascarids (*Ascaris suum*, *Toxocara canis*, *Baylisascaris procyonis*), the spores that colonize the animal feces germinate out a mycelium which penetrates the eggshell, invades the interior, and damages both the eggshell and the embryo [84,85]. Furthermore, it can survive in the digestive tracts of animals without loss of biological activity, thus providing a very helpful tool to prevent infection by ascarids among pasturing animals [22].

The use of independent predatory and ovicidal fungi is effective for the treatment of GT parasitic nematodes in a variety of domestic animals (including sheep, cattle, goats, horses, pigs, and chickens). For some fungi, such as *Po. chlamydosporia* and *Pa. lilacinus*, although good results have been achieved in vitro, few in vivo studies have been performed due to the lack of a reliable approach for administering a standard dose [86]. Differences in the NF trapping efficiency of nematodes are universal among different species of the same genera and different isolated strains of the same species (Table 1 and Table 2). Two factors account for these differences: (1) Internal factors include the cuticular nature of parasitic nematodes and the antigenic variations in the different species of nematodes or different isolates of the same species of fungus [87]. (2) Extrinsic factors include the number of nematodes available and environmental factors, for example, a low density of nematodes being insufficient to stimulate NF to produce trapping structures, resulting in a low predation efficiency of NF [87]. The shaded condition is conducive to the development of fungi and the germination of spores [14,88,89]. Thus, NF show variable effects for controling nematodes.

As shown above, the biggest difference between predatory and ovicidal fungi is that the former target larvae while the latter target eggs, cysts, and nematode females [90,91]. When these two types of fungi are used separately, it is inevitable that either the eggs or larvae will escape capture. With the goal of complementing the advantages and promoting the efficiency of hunting nematodes, some researchers have evaluated joint applications, including the combined use of two biological controls or a mix of chemical and biological controls, on GINs in domestic animals (Table 3). To date, the mechanisms of the synergistic interactions among them are not known, but in some cases the strategy seems to have an additive effect.

## 3. Potential New Strategies of Biological Control

The use of a combination of biological controls or mixed biological and chemical controls may reduce the flaws evident in individual administration, and it may even enhance fungal predation ability [41]. However, there are compatibilities or incompatibilities (the combined agents can produce compounds that inhibit each other in a joint application) among some fungi, predators and compounds, and an incompatibility can seriously prevent the combined strategy from controlling nematodes [21,100,101].

### 3.1. Coadministration of Fungi with Fungi

According to the current studies, the methods of associated application among fungi can be summarized into three forms: a combination of two or three fungi, a combination of different groups of fungi (predatory and ovicidal), and a combination of identical groups of fungi (predatory fungi) (Table 3). In combined administration, these fungi are required to meet two basic requirements: first, they are required to have the ability to pass through the animal’s GT without losing vitality, as if they were administered alone; second, they must not inhibit each other’s growth [100]. These are two basic prerequisites to realize additive effects. To date, the selected fungi are considered effective species that have previously been proven to be able to prey on nematodes when used alone, so they generally satisfy the first prerequisite, while the second needs to be reassessed.

The associations between identical groups of fungi (*D. flagrans* + *Mo. thaumasium*) or different groups of fungi (*A. cladode* + *Po. chlmydosporia*, *D. flagrans + Po. chlamydosporia*, *Mo. thaumasium* + *Po. chlamydosporia*) have been shown to have synergistic effects, which significantly enhance predation efficiency compared to using a single fungus alone [21,78,92,93]. However, not all combined applications have been tested for the compatibility between the fungal participants in the joint action; only *D. flagrans* + *Mo. Thaumasium* and *A. cladode* + *P. chlmydosporia* have presented growth compatibility [21,69,94]. In contrast, some joint strategies have shown antagonistic effects. Luns et al. noted that the pairwise combination of *D. flagrans* with *Mo. thaumasium* or *A. robusta* showed a lower nematicidal percentage than *D. flagrans* alone, and that the combination of all three fungi was the least effective [41]. Similarly, the combination which includes *Clonostachys rosea* has been found to be less efficient than *D. flagrans* alone [95,96]. For *D. flagrans* and *A. robusta*, the low predation efficiency was due to the incompatibility of the two fungi [41]. In addition, although some studies have reported that joint application was effective, these strategies have not been proven to have additive effects [97,98,99]. Therefore, the usefulness of these strategies needs to be examined closely if their predation efficiency cannot exceed the effect of a single-fungus treatment.

It seems as if a combination of different groups of fungi may be more effective than a combination of the same group of fungi, based on the differences in the predation targets of predatory and ovicidal fungi, but this is only a guess. It stands to reason that comparisons between combined applications are restricted because (1) different experimental methods or materials (strains and nematode species) and different test targets (the reduction of L3 in feces, the mean number of eggs per gram of feces (EPG) or the number of infective larvae/kg of dry matter (L3/kg D.M.), etc.) are generally used [74,99], and (2) the convincing cases are rare; when these variables are identical, the results of individual cases are contrary to the hypotheses, e.g., *D. flagrans* + *Mo. Thaumasium* being more effective than *D. flagrans* + *Po. Chlamydosporia* [94]. Therefore, based on the results of the current study, it is impossible to know which fungal combination form works better. Nevertheless, it is absolutely necessary to consider whether there is compatibility between the collaborators before choosing strains. This compatibility can be verified by determining whether zones of inhibition appear in a co-culture of fungi on the same plate [90].

### 3.2. Coadministration of Fungi with Compounds

The combination of chemical controls and NF involves chemical anthelmintic drugs and organic compounds, and the combination of these two kinds of compounds with NF has different effects. Among them, the combination between chemical drugs and NF seems to be effective, because the former acts on nematodes in animals, whereas the latter acts on free-living nematodes in pasture [19,102]. From the perspective of the predation target, this combination could disrupt the whole life cycle of nematodes, but there have been relatively few successful cases of doing so. However, organic compounds could act as efficient vehicles in the topical administration of NF [103,104].

Reports of synergistic effects for the simultaneous administration of chemical drugs and NF are scarce. Vilela et al. reported that the combination of *D. flagrans* and levamisole hydrochloride was feasible, with a significantly lower EPG recorded for their combination than for exclusive administration of the chemical drug [105]. In this regard, Wang et al. similarly noted that *D. flagrans* had strong tolerance to levamisole hydrochloride [106]. Nevertheless, most studies suggest an antagonistic effect between chemical drugs and NF. For example, the antiparasitic compounds albendazole, ivermectin, and levamisole have been found to have a negative impact on the viability of *A. oligospora*, *Pa. lilacinus D. flagrans*, and *Arthrobotrys* sp. [107]. *D. flagrans* has been found to have high susceptibility to tested drugs, including fenbendazole, thiabendazole and ivermectin, carbendazim, and difenoconazole [106]. In vivo tests also confirmed that chemical drugs inhibited the activity of *D. flagrans* spores when used in combination with albendazole [108].

On the other hand, numerous studies have incorporated fungi into a sodium alginate matrix and produced edible pellets, which helps fungi overcome undesirable factors to successfully pass through animal GTs and contributes to the long-term preservation of spores [27,99,103]. Dimethyl sulfoxide as a permeabilizer and mineral oil as an adjuvant assisted in an excellent penetration and adhesion of conidia to the nematode epidermis [109,110]. Importantly, this method presented a high affinity for conidia fungi and effectively acted on *Rhabditis* spp. [104]. These findings were all confirmed under laboratory conditions, except for the compatibility of fungi, which was confirmed with an antiparasitic in vivo test [108,111].

The above results indicate that not all chemical compounds interfere with fungal activity, making their future combined use possible.

## 4. Concluding Remarks

The independent use of a representative species of NF has achieved satisfactory results in controlling GINs, which has stimulated human interest in discovering novel candidates for nematode-trapping fungi. According to the latest assessment, fungal species richness has reached 12 million globally [112], but only approximately 1.2% (140,000) species have been described [113]. However, there are only around 200 species of nematode-trapping fungi with the ability to prey on free-living stage nematodes, accounting for one thousandth of the known fungal population [114]; the number of fungal species that remain to be described leaves great potential for the discovery of novel NF. Furthermore, the ability to capture GINs is different for different species of NF. *D. flagrans* and *A. robusta*, for example, have a significant difference in their ability to reduce the number of GINs; Oliveira et al. believe that it is essential to test the nematicidal ability of each species separately [48]. From the perspective of predatory characteristics, in vivo and in vitro tests need to be continuously performed to verify the efficacy of specific nematophagous fungal species for GIN treatment. Moreover, environmental factors (temperature, humidity, and altitude) have an impact on NF capture of GINs, since each species has its own environmental adaptation ability [48]. Thus, some scholars have suggested isolating potential candidates from the isolates of NF which root in animal feces on local pastures [50,51]. This method makes it easier to obtain potential candidate strains adapted to local climatic conditions, and would reduce invasion by alien species.

Related cases of associated applications have shown that this new method is promising in GIN control, and it has certain practical and research value in the future. Differences in predation efficiency for identical groups of NF, and the different mechanisms of the different groups of NF, may contribute to the positive effects of combinations of fungi. Although resistance is quickly developed to chemical drugs, they are more effective at killing nematodes than fungi, so the combination of the two could also reduce resistance and enhance nematicidal efficiency. Further studies are still needed to establish the pattern of combining NF with chemical drugs, such as the compatibility, appropriate dose, and time [104,106].

## Figures and Tables

**Table 1:** In vivo tests and in vitro nematicidal tests with nematophagous fungi of the genera *Duddingtonia*, *Arthrobotrys*, and *Monacrosporium* on the gastrointestinal nematodes of domestic animals.

Fungi	GIN Species	Dose	Nematicidal Activities		Reference
			In Vivo Test	In Vitro Test	
*A. cladodes*	*Haemonchus* sp., *Cooperia* sp., *Oesophagostomum* sp. (cattle)	1g pellets/10 kg BW, twice a week	52–59%	68.7%	[48,49]
*A. oligospora*	*H. contortus*, *T. colubriformis* (sheep)	5 × 10^5^ spores/kg BW; 2 mL fungal suspension	53.88–97.26%	90–99.99%	[50]
*A. conoides*, *A. sinense*	trichostrongylides (sheep)	5 × 10^5^ spores/kg BW; 2 mL fungal suspension	37.84–78.64%, 54.49–86.93%	80.00–97.41%, 97.02–98.49%	[18]
*A. superba*	*Haemonchus contortus* (sheep)	5 × 10^5^ spores/kg BW; 2 mL fungal suspension	83.79%	86.48–97.69%	[19]
*A. musiformis*, *A. robusta*	trichostrongylides (goat)	5 × 10^5^ spores/kg BW; 2 mL fungal suspension	47.60–55.93%, 41.96–66.97%	97.71–99.98%, 97.99–99.95%	[51]
*D. flagrans*	cyathostomins (horse)	6 × 10^5^ chlamydospores/kg, BW for 21 days	37.24–98.62%		[37]
*D. flagrans*	*Haemonchus contortus* (sheep)	5 × 10^5^ spores/kg BW; 2 mL fungal suspension	55.15–98.82%	62.12–99.88%	[43]
*D. flagrans*	*Strongyloides papillosus* (sheep)	1g Bioverm® (10^5^ chlamydospores/g)		91.5%	[17,52]
*D. flagrans*	*Haemonchus contortus* *Trichostrongylus colubriformis*, *Teladorsagia circumcincta*, *Bunostomum ovina*, *Chabertia ovina* (sheep)	5 × 10^5^ spores/kg BW, twice a week	85.4%, 87.5%, 90%, 81.0%, 71.4%,		[24]
*D. flagrans*	*Haemonchus* spp., *Trichostrongylus* spp., *Oesophagostomum* sp. *and Strongyloides* sp. (cattle)	1 g Bioverm® /10 kg BW, (containing 10^5^ chlamydospores)	88.2%,		[47]
*Mo. thaumasium*	cyathostome (horse)	1ml of solution containing 1000 spores, single dose		95%	[53]
*Mo. thaumasium*	*Oxyuris equi* (horse)	Each petri dish contained fungal isolate		69%	[54]
*Mo. thaumasium*	*Haemonchus*, *Trichostrongylus*, *Oesophagostomum*, *and Strongyloides* (sheep)	3 g of pellets/10 kg BW	79%		[55]
*Mo. thaumasium*	The gastrointestinal nematodes (sheep)	100 g pellets (20g fungal mycelia), single dose		93%	[27]
*Mo. thaumasium*	trichostrongylides, *Marshallagia mongolica* (sheep)	5 × 10^5^ spores/kg BW; 2 mL fungal suspension	51.68–88.16%	75.54–99.97%	[19]

Note: trichostrongylides: mainly *H. contortus* and *T. colubriformis*; cyathostomins: the gastrointestinal nematodes of horses; BW: body weight; in the dose column, the administered doses for in vivo tests and in vitro tests are separated using semicolons.

**Table 2:** In vivo tests and in vitro nematicidal tests of the genera *Pochonia*, *Paecilomyces*, and *Mucor* on gastrointestinal nematodes of domestic animals.

Fungi	GIN Species	Dose	In Vivo/Vitro Test	Reference
*Po. chlamydosporia*	*Ascaridia galli*, *Heterakis spp* (chicken)	0.9 × 10^6^ chlamydospores and 5.4 × 10^7^ conidia	75%	[59]
*Po. chlamydosporia*	*Oxyuris equi* (horse)	100g pellets, single does; each Petri dish contained fungal isolate	21.8%/27.2%	[22,54]
*Po. chlamydosporia*	*Oxiuris equi* (horse)	Fungal isolate added gelatin	72%	[60]
*Po. chlamydosporia*	*Anoplocephala perfoliata Eggs* (horse)	Each Petri dish contained fungal isolate	71.17%	[61]
*Po. chlamydosporia*	*Ascaridia galli*, *Heterakis gallinarum* (chicken)	3.3 × 10^6^ conidia/chlamydospores, single dose; subcultures were inoculated in petri dishes	59.9%, 43.2%	[62]
*Po. chlamydosporia*	*Toxocara canis* (dog)	1.0 × 10^5^ chlamydospores, various concentrations	78.5%	[63]
*Po. chlamydosporia*	*Haemonchus*, *Cooperia*, *Oesophagostomum* (bovine)	Each Petri dish contained fungal isolate	87.4%	[64]
*Pa. lilacinus*	*Oxiuris equi* (horse)	Fungal isolate added gelatin	62%	[60]
*Po. chlamydosporia*	*Parascaris equorum* (horse)	Each Petri dish contained fungal isolate	44.9%	[65]
*Pa. lilacinus*	*Toxocara canis* (dog)	Each Petri dish contained fungal isolate	20.0%	[66]
*Pa. lilacinus*	*Ascaridia galli* (chicken) *Toxocara canis* (dog)	1.5 × 10^5^ conidia	15–29%, 4–28%	[67]
*Po. Chlamydosporia*	*Ascaridia galli* (chicken), *Toxocara canis* (dog)	1.5 × 10^5^ conidia	64–86%, 26–67%	[67]
*Mu. circinelloides*	*Ascaris suum* (pig)	The mash with fungal spores (2 kg/ pig/day); 1 × 10^6^ spores	60/53%	[68]
*Mu. circinelloides*	*Parascaris equorum* (horse)	1 mL pellet, 2 × 10^6^ spores/mL	61–67%	[69]

Note: In the dose column, the administered doses for in vivo tests and in vitro tests are separated using semicolons.

**Table 3:** Overview of in vivo and in vitro nematicidal tests of the combined application of representative nematophagous fungi against gastrointestinal nematodes.

Nematophagous Fungus (% Reduction of L3 Numbers)	GIN Species	Comment	References
*A. cladodes* (77.0%) + *Po. chlamydosporia* (66.8%) [86.3%]	*Haemonchus*, *Cooperia*, *Oesophagostomum.* (cattle)	In vitro, synergistic effect	[78]
*D. flagrans* (58.9%) + *Mo. thaumasium* (34%) [83%]	*Haemonchus* sp., *Trichostrongylus* spp., *Strongyloides* sp., *Oesophagostomum* sp. (sheep)	In vitro, synergistic effect	[92,93]
*A. cladodes* (81.73%) + *Po. chlmydosporia* (68.25%) [92.67%]	*Haemonchus*, *Cooperia*, *Oesophagostomum* (cattle)	In vitro, synergistic effect, compatibility	[21]
*D. flagrans* (61.6%) + *Mo. thaumasium* (66.1%) [92.4%]; *D. flagrans*(61.6%) + *Po. chlamydosporia* (73.2%) [86.8%]; *Mo. thaumasium* (66.1%) + *Po. chlamydosporia* (73.2%) [77.3%]	cyathostomin (horse)	In vitro, synergistic effect, compatibility (*D. flagrans* + *Mo.* *thaumasium*)	[69,94]
*D. flagrans* (96.4%) + *Mo. thaumasium* (93.4%) [90.7%]; *D. flagrans* (96.4%) + *A. robusta* [86.3%]; *D. flagran* (96.4%) + *Mo.* *thaumasium* + *A. robusta* [78.3%]	*Cooperia* sp., *Haemonchus*, *Oesophagostomum* (cattle)	In vitro, antagonistic effect, incompatibility (*D. flagrans* + *A. robusta*)	[41]
*D. flagrans* (91.5%) + *Clonostachys rosea* (88.9%) [74.5%]	*Haemonchus contortus* (sheep)	In vitro, antagonistic effect	[95,96]
*A. robusta* + *D. flagrans* [93%] *A. conoides* + *Mo. thaumasium* [98%]	The gastrointestinal nematodes (goat)	In vitro, associated application showed high predatory activity	[97]
*D. flagrans+ Po. chlamydosporia+ A. robusta* [94%, 91.8%]	*Haemonchus* sp., *Cooperia* sp., *Oesophagos-* *tomum* sp. (cattle)	In vitro and vivo, associated application showed high predatory activity	[98]
*D. flagrans* + *Mo. thaumasium* (>80%)	cyathostomin (horse)	In vitro, associated application showed high predatory activity	[99]

Note: () and [], respectively, express the percentage reduction of larvae by individual and associated applications of nematophagous fungi. Additive effect: the effect of associated application was better than that of individual application.

## Data Availability

Not applicable.
